# Artificial Intelligence Analysis of Celiac Disease Using an Autoimmune Discovery Transcriptomic Panel Highlighted Pathogenic Genes including BTLA

**DOI:** 10.3390/healthcare10081550

**Published:** 2022-08-16

**Authors:** Joaquim Carreras

**Affiliations:** Department of Pathology, School of Medicine, Tokai University, 143 Shimokasuya, Isehara 259-1193, Japan; joaquim.carreras@tokai-u.jp; Tel.: +81-0463931121

**Keywords:** celiac disease, gluten-sensitive enteropathy, BTLA, autoimmunity, gene expression, artificial intelligence, machine learning, artificial neural networks, immuno-oncology, immune checkpoint

## Abstract

Celiac disease is a common immune-related inflammatory disease of the small intestine caused by gluten in genetically predisposed individuals. This research is a proof-of-concept exercise focused on using Artificial Intelligence (AI) and an autoimmune discovery gene panel to predict and model celiac disease. Conventional bioinformatics, gene set enrichment analysis (GSEA), and several machine learning and neural network techniques were used on a publicly available dataset (GSE164883). Machine learning and deep learning included C5, logistic regression, Bayesian network, discriminant analysis, KNN algorithm, LSVM, random trees, SVM, Tree-AS, XGBoost linear, XGBoost tree, CHAID, Quest, C&R tree, random forest, and neural network (multilayer perceptron). As a result, the gene panel predicted celiac disease with high accuracy (95–100%). Several pathogenic genes were identified, some of the immune checkpoint and immuno-oncology pathways. They included *CASP3*, *CD86*, *CTLA4*, *FASLG*, *GZMB*, *IFNG*, *IL15RA*, *ITGAX*, *LAG3*, *MMP3*, *MUC1*, *MYD88*, *PRDM1*, *RGS1*, etc. Among them, B and T lymphocyte associated (BTLA, CD272) was highlighted and validated at the protein level by immunohistochemistry in an independent series of cases. Celiac disease was characterized by high BTLA, expressed by inflammatory cells of the lamina propria. In conclusion, artificial intelligence predicted celiac disease using an autoimmune discovery gene panel.

## 1. Introduction

Celiac disease is a frequent type of immune-mediated inflammatory disease of the small intestine. This gluten-sensitive enteropathy is caused by higher sensitivity of the gut and immune system to gluten of the diet and to gluten-related proteins [1].

The pathogenesis of celiac disease depends on genetic factors and mucosal immune response. This immune disorder occurs in genetically predisposed patients after induction by an environmental factor, which is gluten in the diet, found in cereals. More than 99% of the patients have *HLA DR3-DQ2* and/or the *DR4-DQ8* [2,3,4], but other non-HLA locus genes may also be involved in the disease pathogenesis, such as *TNFAIP3 (A20)*, *REL*, *NKG2D*, *MICA*, *CTLA4*, *MMP3*, *MIF*, and etcetera [5,6,7,8,9,10,11,12,13,14,15]. Celiac disease is associated with several autoimmune disorders, such as type 1 diabetes mellitus and autoimmune thyroid disease [16,17]. The mucosal immune response also participates in the disease pathogenesis. An inflammatory reaction develops in response to gliadin fractions, and a result there is inflammation of the lamina propria and epithelium, with disruption of the epithelial layer and villous atrophy. Both the innate and adaptive immune responses are activated in celiac disease, including gliadin reactive T cells, autoantibodies, intraepithelial lymphocytes, macrophages, monocytes, and dendritic cells.

A detailed description of the pathogenesis of celiac disease is shown in Table 1.

Celiac disease has an estimated prevalence of 1% in the general population based on serologic studies, although in many cases the disease is asymptomatic [52,53]. The most relevant clinical manifestation is due to malabsorption, and includes diarrhea, weight loss, anemia, and other metabolic disturbances. Of note is that celiac disease can have diverse extraintestinal presentations such as delayed puberty, hepatitis, iron-deficiency anemia, arthralgia and arthritis, peripheral neuropathy, epilepsy and seizures, cerebellar ataxia, and dermatitis herpetiformis (among others) [1,29]. The diagnosis is made by a combination of clinical signs and symptoms, serology testing, and small intestine biopsy. Additional diagnostic tools include HLA typing, quantification of inflammatory cells in the small intestine biopsy such as increased CD3-positive lymphocytes in the villus tips or the quantification of intra-epithelial lymphocytes (IELs), and detection of TG2-targeted celiac IgA isotype autoantibodies in the intestinal mucosa, and detection of gluten-specific T cells in the circulation by ELISPOT [29].

Celiac disease has associated conditions including selective IgA deficiency, autoimmune disease, gastrointestinal disease (reflux disease, eosinophil esophagitis, inflammatory bowel disease, microscopic colitis, liver disease, and pancreatitis), menstrual and reproductive issues, idiopathic pulmonary hemosiderosis, and cardiovascular and kidney diseases [1].

Celiac disease is associated with several autoimmune diseases including diabetes mellitus type 1 [54,55,56,57], autoimmune thyroid disease (hypothyroidism) [58,59], and atopic dermatitis [60,61]. Other manifestations related to serological autoantibodies includes neurological disorders (peripheral neuropathy and ataxia) [62], and neurodegeneration via apoptosis [63].

Patients with untreated celiac disease are at increased risk of lymphoma and gastrointestinal cancer [1]. Patients with refractory celiac disease type II may be associated with enteropathy-associated T-cell lymphoma (EATL) [64,65,66,67,68,69].

Due to the clinical relevance of this disease, a better understanding of the pathogenesis is needed, and using non-linear analysis may provide a different approach. This research was a proof-of-concept exercise to determine whether artificial intelligence analysis was a feasible approach to model celiac disease using an autoimmune discovery gene panel.

## 2. Materials and Methods

### 2.1. Celiac Disease GSE164883 Dataset

A suitable gene expression dataset was searched at the Gene Expression Omnibus (GEO) database search engine of the National Library of Medicine, National Center for Biotechnology Information (NIH): https://www.ncbi.nlm.nih.gov/ (last accessed 11 July 2022). A public dataset from 24 March 2021, the GSE164883, was selected and downloaded [70]. This dataset, published by Dr. Worf J et al., includes a high-resolution analysis of transcriptomes obtained from 48 duodenal biopsies of 26 children and adolescents diagnosed with celiac disease, and 22 children without celiac disease as controls. Biopsies were obtained from the descending duodenum and snap frozen using liquid nitrogen. After homogenization (TissueLyzer, Qiagen, Hilden, Germany), total RNA was extracted using AllPrep^®^ DNA/RNA Microkit (Qiagen). The Illumina HumanHT-12 V4.0 gene expression beadchip was used [70].

The clinical characteristics were as follows: in the celiac disease group, the age ranged from 3 to 17 years, with a median of 9.5, and a mean of 9.0 ± 4.5. Based on the Marsh classification, the stage was 3 in all cases, A in 6 of 26 (23.1%), B in 13 of 26 (50%), and C in 7 of 26 (26.9%). In the control group, the age ranged from 1 to 17 years, with a median of 12.5, and a mean of 11.4 ± 4.8 years. Based on the Marsh classification, 0 in 19 of 22 (86.4%), and 1 in 3 of 22 (13.6%) [70].

### 2.2. GEOR Analysis

The GEO2R software was used to compare two groups of samples (celiac disease versus control) to identify genes that were differentially expressed across experimental conditions. The adjustment to the *p* values was Benjamini & Hochberg (false discovery rate), apply transformation to the data (auto-detect), no application of limma precision weights (wooma), no force normalization. The significance level cut-off was set at 0.05. The software runs in R 3.2.2 (R Foundation for Statistical Computing, Vienna, Austria), Biobase 2.30.0, GEOquery 2.40.0, and limma 3.26.8.

### 2.3. Transcriptome Panels

The autoimmune discovery transcriptome panel contains 755 genes that are either closely associated with germline variants across nine different autoimmune diseases or are relevant to the immune response. The nine autoimmune diseases are celiac disease (n = 249), ulcerative colitis (n = 201) and Crohn’s disease (i.e., inflammatory bowel disease, n = 253), multiple sclerosis (n = 104), rheumatoid arthritis (n = 95), systemic lupus erythematosus (n = 55), type 1 diabetes mellitus (n = 44), psoriasis (n = 48), and ankylosing spondylitis (n = 43). Of note, some genes overlap in different categories. That panel was curated from studies that were available from the ImmunoBase database or from genome-wide association (GWAS) studies. The database can be explored at the following link: https://genetics.opentargets.org/immunobase; https://www.opentargets.org/; https://docs.google.com/spreadsheets/d/1YYbxC1NhtbYuBYe2gYZNcxO0a0S4oTxHfoYtZrqKsrM/edit#gid=1589938306 (accessed on 11 July 2022). The list of 755 genes can be accessed at the following link: https://doi.org/10.5281/zenodo.6976192 (accessed on 9 August 2022).

Additional panels were also included in the analysis, including the metabolic pathways (n = 751 genes), immune exhaustion (n = 803), human inflammation (n = 250), host response (n = 790), autoimmune (n = 756), organ transplantation (n = 765), cancer transcriptomic atlas (n = 1794), pan-cancer human (n = 755), pan-cancer immune profiling (n = 730), pan-cancer progression (n = 742), and pan-cancer pathways (n = 730). These panels were previously used in the mantle cell lymphoma and artificial intelligence project [71].

### 2.4. Gene Set Enrichment Analysis (GSEA)

The GSEA software (GSEA v4.2.3) was downloaded from the Broad Institute, Inc., Massachusetts Institute of Technology, and Regents of the University of California webpage: http://www.gsea-msigdb.org/gsea/index.jsp (accessed on 11 July 2022).

The following molecular signatures, divided into nine major collections of gene sets (database v7.5.1), were downloaded: H (hallmark), C1 (positional), C2 (curated), C3 (regulatory target), C4 (computational), C5 (ontology), C6 (oncogenic signature), C7 (immunologic signature), and C8 (cell type signature gene sets).

Four types of files were created, the gene expression dataset (*gct*), the phenotype labels (*cls*), the gene sets (*gmx*), and the annotations (*chip*). As parameters, the number of permutations was set at 1000. Phenotype labels: celiac disease versus control. Collapse to gene symbols using max. probe. Permutation type: phenotype. Enrichment statistic: weighted. Metric for ranking genes: sinal2noise. Gene list sorting mode: descending. Normalization mode: meandiv. Seed of permutation: timestamp. Randomization mode: no balance. Of note, the autoimmune discovery panel and the other additional panels were also coded into *gmx* gene sets.

### 2.5. Statistical Analyses

All analyses were performed using a desktop equipped with the following hardware: AMD Ryzen^TM^ 9 5900X processor (12 CPU cores, L2 cache 6 MB, L3 cache 64 MB), an Nvidia GEFORCE RTX 3060 Ti graphic card, and 16.0 GB of RAM.

IBM SPSS version 27.0.1.0 (64-bit edition) was used for the basic statistical analyses (IBM Corporation, New Orchard Road Armonk, New York, NY, USA). Additionally, several software applications were used for acquisition, processing, analysis, and validation/confirmation of results. The software included Microsoft excel 2016 (Microsoft Corporation, One Microsoft Way, Redmond, WA, USA), EditPad Lite (Just Great Software Co., Ltd., Rawai Phuket, Thailand), GSEA v4.2.3 (UC San Diego, Broad Institute, Merkin Building, 415 Main St., Cambridge, MA, USA), JMP Pro 14 (JMP Statistical Discovery LLC, SAS Institute Japan Ltd., Roppongi, Minato-ku, Tokyo, Japan), Minitab 21 (Minitab, LLC, State College, PA, USA), IBM SPSS modeler 18 (IBM), and RapidMiner Studio 9 (RapidMiner, Inc., Boston, MA, USA). GEO2R ran on R 3.2.3, Biobase 2.30.0, GEOquery 2.40.0, and limma 3.26.8. All the analyses were performed as previously described in our previous publications [72,73,74,75,76,77,78,79,80,81]. The multilayer perceptron analysis is described in references [72,76,78]. Immunohistochemical procedures are described in references [73,74,75,77]. Machine learning techniques are shown in references [75,79,80]. The method of analysis of this research is equivalent to the one recently published in ulcerative colitis [81].

### 2.6. Immunohistochemical Analysis of BTLA in an Independent Series

*BTLA* was analyzed at protein level by immunohistochemistry using an automated stainer (Leica BOND-MAX) following the manufacturer’s instructions. The primary antibody was obtained from Dr. Giovanna Roncador (Monoclonal Antibodies Laboratory, Spanish National Cancer Research Institute, CNIO, Madrid, Spain). The primary antibody, mouse monoclonal, targeted BTLA (B and T lymphocyte associated protein), clone name FLO67B. The antigen used was BTLA-HIS recombinant protein (full-length protein without signal peptide 25–289aa). IgG1 isotype. Species reactivity, human. Localization, membrane/cytoplasm. Positive control, tonsil. The recommended dilution, 1:5 (supernatant) or 1:100 (purified antibody, 1 mg/mL). Antigen retrieval, 20 min ER2 (Tris-EDTA). Antibody incubation, 15 min. The detection system, BOND Polymer Refine Detection (BOND-MAX, Leica).

Immunohistochemistry was performed in 16 celiac disease patients (57 biopsies), and 16 control cases (16 small intestine biopsies). The cases were selected from the Department of Pathology, Hospital Clinic of Barcelona, Spain. The cases were diagnosed in patients with positive celiac serology, based on histological criteria using biopsies of the small intestine: the presence of increased intraepithelial lymphocytes with crypt hyperplasia (Marsh type 2) or with villous atrophy (Marsh type 3) (Appendix A, Table A1).

## 3. Results

Summary of the results.

- A conventional analysis using GEO2R highlighted the genes differentially expressed between celiac disease and control.
- Gene set enrichment analysis (GSEA) identified the gene sets (pathways) that were associated with celiac disease, including the autoimmune discovery panel.
- Several Machine learning and artificial neural network analyses predicted celiac disease using the autoimmune discovery panel with high accuracy.
- Celiac disease was characterized by high expression of *BTLA* both at the gene expression level, and at protein level by immunohistochemistry in a validation series.

### 3.1. Gene Expression Analysis Using the GEO2R Software

**Figure 1:**
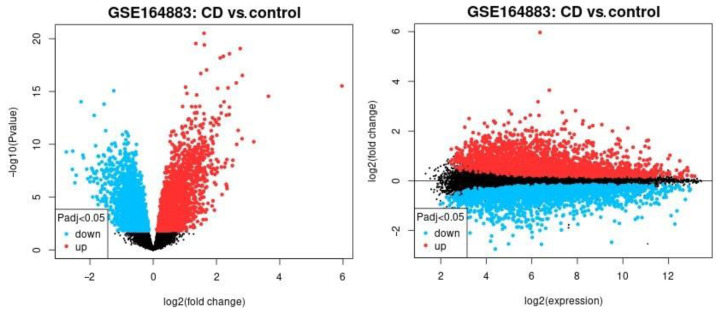
The differential gene expression between celiac disease and control cases. The gene expression of the groups of celiac disease and control samples were compared using GEO2R software. The up-regulated genes are highlighted in red, the down-regulated in blue, and the non–significant in black. Left, the volcano plot. Right, mean difference plot.

The differential gene expression across celiac disease and control cases was analyzed using a conventional method (NCBI GEO2R software), and the result is shown in Figure 1. In this analysis, all the genes of the Illumina HumanHT-12 V4.0 gene expression beadchip were used to explore broadly the expression of celiac disease. The most significantly up-regulated genes in celiac disease were *TAP1*, *HLA-E*, *HCP5*, *STAT1*, *GBP1*, *STAT1*, *LOC100419583*, and *GBP4* and the down-regulated ones were *IDS*, *PKIB*, *FBXO2*, *OXT*, and *ADI1*.

### 3.2. Gene Set Enrichment Analysis (GSEA)

To improve the analysis of GEO2R software, a pathway analysis was performed using the gene set enrichment analysis (GSEA). GSEA is a computational method that determines whether a priori set of genes shows statistically significant, concordant differences between two groups.

The analysis using all gene sets of all collections of the Molecular Signatures Database (MSigDB version 7.5.1) was successful. In the nine major collections, a total of 5600 sets were significantly enriched at a nominal *p* value of <1%.

**Figure 2:**
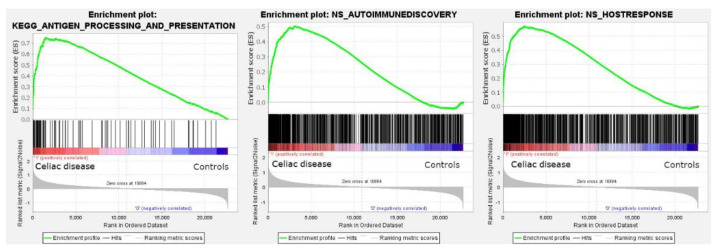
Gene set enrichment analysis (GSEA). GSEA analysis was performed to identify gene sets (i.e., pathways) associated with celiac disease. All the sets of the nine major collections of the Molecular Signatures Databases were tested and 5600 sets were significantly enriched at a nominal *p* value of <1%. Among them, the antigen processing and presentation is highlighted (left). The autoimmune discovery transcriptome panel and additional panels were also tested, and showed an enrichment (association) toward celiac disease (autoimmune discovery panel, center; host immune response panel, right).

Among the C2 curated gene sets, one of the most significant was the M16004 KEGG antigen processing and presentation set (in the leading edge of the core enrichment, *TAP1*, *HLA-E*, *RFX5*, *IFI30*, *CD8A*, etc.) (Figure 2). Other relevant pathways within the C2 set were the M15615 interferon gamma response (*IFNG*), M543, M7963, and M16647 cell cycle, M15381 TCR signaling, M11884 antigen response, and M1060 cytokine signaling.

The GSEA analysis using the autoimmune discovery panel and additional panels such as the host immune response were also statistically significant and enriched the celiac disease group (Figure 2). The most significant genes at the leading edge of the core enrichment of the autoimmune discovery panel were *STAT1*, *GBP1*, *IFNG*, *IRF1*, *RIPK2*, *CXCL10*, *CXCR6*, *BATF*, *ITGAL*, and *GFI*. Additional markers relevant to the pathogenesis (with the immune microenvironment) of celiac disease were also found within the core enrichment, including *LAG3*, *MICB*, *RUNX3*, *CASP3*, *IL15RA*, *FASLG*, *CTLA4*, *IL10RA*, *GZMA*, *RGS1*, *IRF4*, *XBP1*, *CD69*, *NFKB1*, *BTLA*, *TIGIT*, *ICOS*, *CD86*, *ITGAX*, *CD274*, *TNFAIP3*, *MMP3*, *MIF*, *BTK*, and *MYD88*.

### 3.3. Artificial Intelligence Analysis

**Figure 3:**
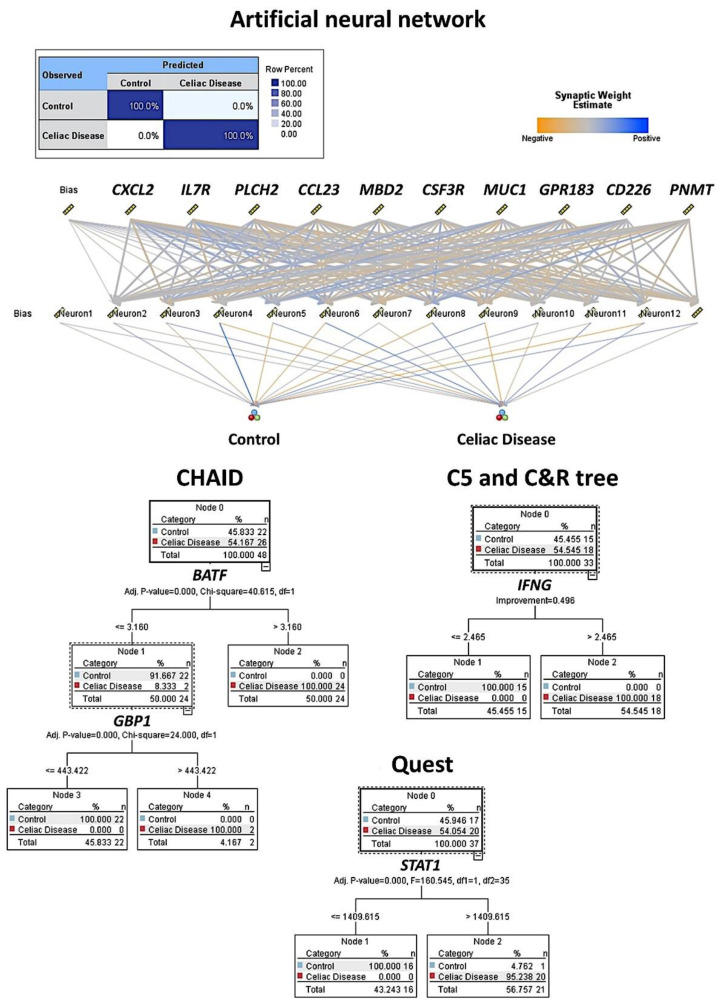
Machine learning and artificial neural network analysis for predicting celiac disease. This figure shows the results of the modeling of celiac disease using an artificial neural network, CHAID, C5, C & R, and Quest decision trees. The overall accuracy ranged from 96% to 100% using as predictors the gene expression (transcriptomic) data of the autoimmune discovery panel.

**Figure 4:**
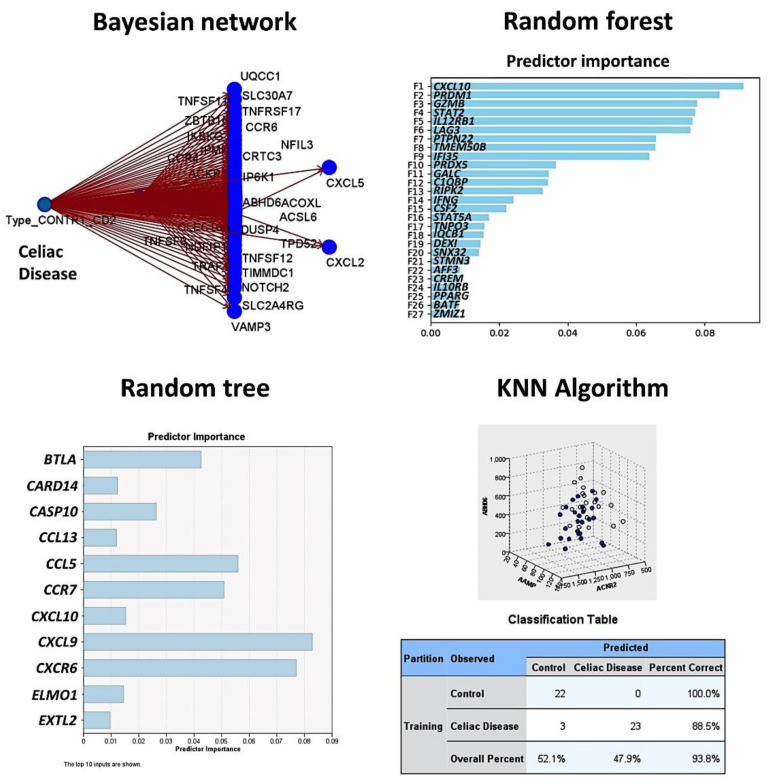
Prediction of celiac disease using the Bayesian network, random forest and tree, and the KNN algorithm. This figure shows the results of the modeling of celiac disease using the autoimmune discovery panel. The Bayesian network shows the genes (nodes) and the probabilistic, or conditional, independencies between them. The causal relationships may be represented, but the links (arcs) of the network do not necessarily represent direct cause and effect. The random forest plot and tree show the genes of the model, ranked according to their predicted importance. The KNN chart is a lower-dimensional projection of the predictor space, which contains 737 predictors (genes of the autoimmune discovery panel).

Based on the autoimmune discovery panel, celiac disease prediction and modeling was performed using several machine learning and artificial neural networks. In total, 737 genes from the panel were used as predictors (inputs, fields) of celiac disease (dependent variable: celiac disease versus control). Among the 15 different techniques, the overall accuracy for prediction was 100% in 11 (73%), 96% in 2 (13%), 86% in 1 (7%), and 0% in 1 (7%) (Table 2 and Table 3, Figure 3 and Figure 4). Of note is that each type of analysis used a specific number of genes, and the type of information and data interpretation was different. Generally, all methods managed to highlight genes characteristic of celiac disease, and some genes were selected in different models. The relevant genes that were identified and that play a role in the pathogenesis of celiac disease were *IFNG*, *CASP3*, *MIF*, *PRDM1*, *GZMB*, *LAG3*, *MUC1*, *CD226*, *BTLA*, and *BTK* (among others).

The artificial neural network was a multilayer perceptron. The network architecture had three layers. The input layer included the predictors (737 nodes, one for each gene). The hidden layer had 12 neurons (the number of units was automatically computed). The stopping rule used was the minimum error ratio achieved. The output layer had two nodes (celiac disease and control). Other build options were the following: overfit prevention set (30%), replicate results (true), random seed (229176228), and missing values in predictors (delete listwise). The accuracy of the model was 100%.

The build settings for each technique are available upon request.

### 3.4. Differential Gene Expression of BTLA between Celiac Disease and Control Samples

**Figure 5:**
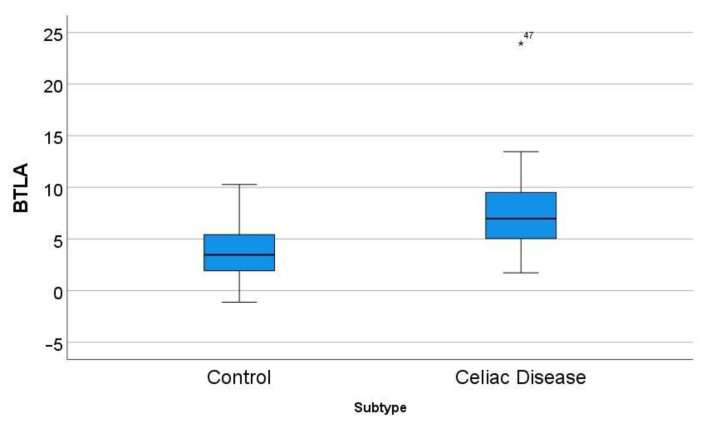
Differential gene expression of *BTLA* between celiac disease and control in the series GSE164883. A direct comparison was statistically significant: 7.8 ± 4.4 vs. 3.7 ± 2.8 (*p* < 0.001). The icon “*” corresponds to a far outlier, and the number “47” is the case number (i.e. the *BTLA* expression value for case 47 was 23.94).

In the GSE164883, *BTLA* was identified as a relevant marker in several techniques, including gene set enrichment analysis (GSEA), logistic regression, random trees, and artificial neural networks. A direct comparison of the gene expression of *BTLA* between celiac disease and control was statistically significant: 7.8 ± 4.4 vs. 3.7 ± 2.8 (*p* < 0.001) (Figure 5).

### 3.5. Validation of BTLA by Immunohistochemistry in an Independent Series

*BTLA* was analyzed at protein level by immunohistochemistry in an independent series of 16 celiac disease patients (with a total of 57 biopsies) and 16 small intestine controls (16 biopsies). The digital images of BTLA are uploaded to zenodo platform as a zip file (https://doi.org/10.5281/zenodo.6837120, accessed on 13 July 2022) (see Supplementary Materials). In the celiac disease cases, four biopsies were excluded from the analysis as BTLA expression was completely absent (0% of positive cells in the inflammatory infiltrate of the lamina propria) without the presence of internal controls.

**Figure 6:**
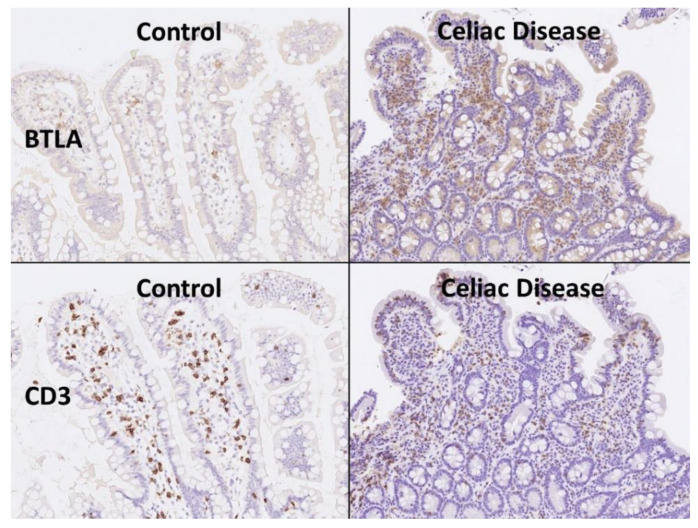
BTLA protein expression by immunohistochemistry. Celiac disease cases were characterized by chronic inflammation of the lamina propria that was BTLA-positive. Using CD3 the T-cell lymphocytes are highlighted, including the higher presence of intraepitheal lymphocytes (IELs that characterize celiac disease). BTLA, B and T lymphocyte attenuator, is an inhibitory receptor with similarities to CTLA and PD-1. BTLA-deficient mice have increased specific antibody responses and enhanced sensitivity to experimental autoimmune encephalomyelitis (Uniprot).

**Figure 7:**
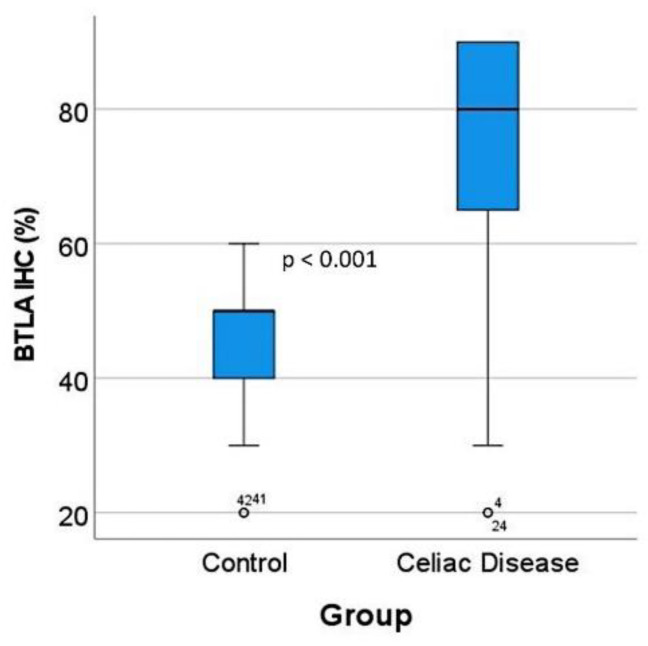
BTLA protein expression by immunohistochemistry in the validation series. After BTLA immunohistochemistry and quantification, celiac disease cases were characterized by high BTLA protein expression (*p* < 0.001). The outliers are marked with a circle, next to the icon there is a number that corresponds to the case number.

**Figure 8:**
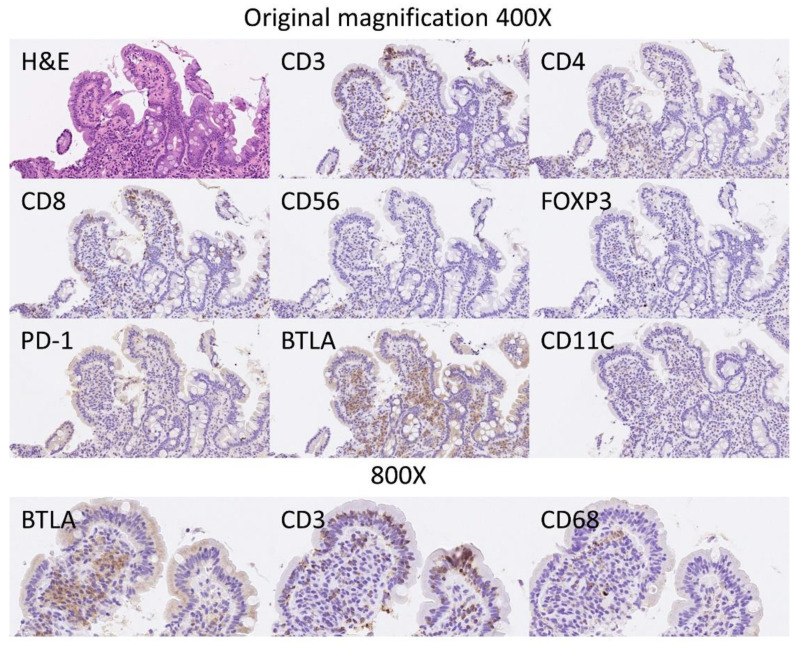
BTLA protein expression by immunohistochemistry in relationship with other markers. Hematoxylin & Eosin staining confirmed the histological diagnosis of celiac disease, with increased intraepithelial lymphocytes (IELs) and with villous atrophy. The IELs were CD3+, CD4−, CD8+ and CD56−. Scarce FOXP3+regulatory T lymphocytes (Tregs) could be identified in the lamina propria. PD-1 staining was negative. The staining with BTLA was high in the lamina propria, and had a pattern of B lymphocytes. BTLA, B and T lymphocyte attenuator, is an inhibitory receptor with similarities to CTLA and PD-1. BTLA-deficient mice have increased specific antibody responses and enhanced sensitivity to experimental autoimmune encephalomyelitis (Uniprot).

The BTLA protein expression was evaluated in the inflammatory infiltrate of the lamina propria, and the percentage of positive cells estimated. The results showed that celiac disease was characterized by a higher frequency of BTLA-positive cells than controls: 70% ± 22.2 vs. 45.6% ± 12.6, respectively (*p* < 0.001) (Figure 6 and Figure 7). Additional immunophenotipic characterization is shown in Figure 8, which confirmed that BTLA mainly identified B lymphocytes of the lamina propria.

### 3.6. Differential Gene Expression of LAG3 between Celiac Disease and Control Samples

**Figure 9:**
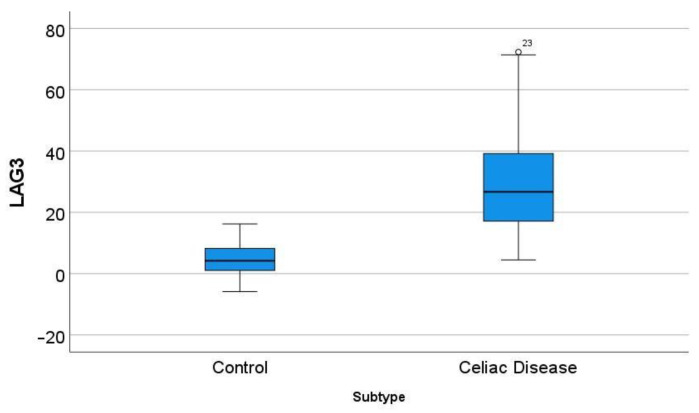
Differential gene expression of *LAG3* between celiac disease and control in the series GSE164883. A direct comparison was statistically significant: 30.7 ± 17.9 vs. 4.6 ± 4.9 (*p* < 0.001). The outliers are marked with a circle, next to the icon there is a number that corresponds to the case number.

In the GSE164883, *LAG3* was identified as a relevant marker in several techniques, including gene set enrichment analysis (GSEA), random forest, and artificial neural networks. A direct comparison of the gene expression of *LAG3* between celiac disease and control was statistically significant: 30.7 ± 17.9 vs. 4.6 ± 4.9 (*p* < 0.001) (Figure 9).

This marker was also analyzed by immunohistochemistry. Despite the fact that the external and internal histological controls were positive, no staining of LAG3 was found in the lamina propria of celiac disease cases.

## 4. Discussion

This research performed a comprehensive analysis of celiac disease. First, artificial intelligence analysis predicted and modeled celiac disease using gene expression data, and as a result, several pathogenic candidates were highlighted. Additionally, other known pathogenic players were identified, which proved the validity of this type of proof-of-concept approach. Then, one of the highlighted markers was validated at protein level by immunohistochemistry in an independent series. BTLA was identified as a maker of the lymphocytes that form part of the chronic inflammatory infiltrate of the lamina propria.

**Figure 10:**
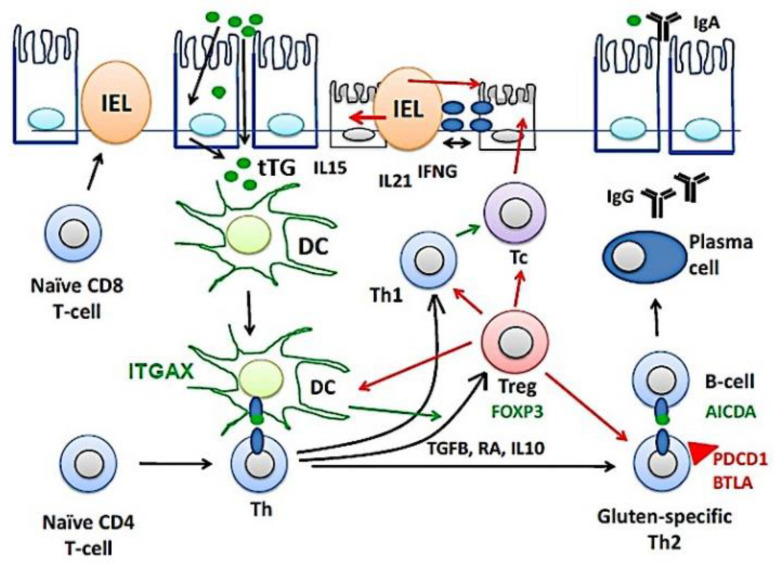
The pathogenesis of celiac disease. The pathogenesis of celiac disease depends on genetic susceptibility and environmental factors (dietary gluten, gliadin). An abnormal immune response in the lamina propria will lead to the chronic inflammation of the mucosa, increased intraepithelial lymphocytes (IELs), and disruption of the epithelial layer. BTLA, B and T lymphocyte associated; DC, dendritic cell; Th, T-helper lymphocyte; IFNG, interferon gamma; Tc, cytotoxic T lymphocyte; tTG, tissue transglutaminase; Treg, regulatory T lymphocyte.

Figure 10 shows a part of the pathogenesis of celiac disease. Despite harboring the genetic susceptibility and gluten (gliadin) consumption, in most cases the disease is latent and histologically normal. Nevertheless, in around 1% of the cases the patients are diagnosed because of clear clinical symptoms and histological criteria [1,2,3,4,5,6,7,8,9,10,11,12,13,14]. The immunological model suggests that gluten-specific CD4+T-cells and cytotoxic intraepithelial T lymphocytes (IEL) play a key role in the development of celiac disease [82,83,84], as defined by the presence of anti-TG2 antibodies and villous atrophy [85]. TGFB, retinoic acid (RA) and IL10, mucosal immune regulatory molecules, regulate the lamina propria inflammation by inducing the generation of regulatory T lymphocytes (Treg), a process regulated by CD11C (ITGAX)-positive dendritic cells (DC) [1,2,3,4,5,6,7,8,9,10,11,12,13,14]. Thus, Tregs will increase as a response to dampen the activation of effector mechanisms, both innate and humoral that destroy the mucosa [86]. Additionally, part of the epithelial damage is mediated by cytotoxic IELs that express activating NK cell receptors (mediated by IL15), which recognize stress- and inflammation-induced ligands on intestinal epithelial cells [1,2,3,4,5,6,7,8,9,10,11,12,13,14]. In this research, celiac disease was characterized by increased expression of BTLA in the lamina propria. The immunohistochemical pattern was a mixture of T and B lymphocytes. This result suggests that the immune checkpoint mechanism of BTLA is up-regulated during disease, and highlights the importance of suppression mechanisms. BTLA, B and T lymphocyte attenuator, is an inhibitory receptor with similarities to CTLA and PD-1. BTLA-deficient mice have increased specific antibody responses and enhanced sensitivity to experimental autoimmune encephalomyelitis (Uniprot).

Machine learning is a branch of artificial intelligence (AI) that specializes in the application of data and algorithms to simulate the way that humans learn, gradually improving its accuracy [87,88,89]. Presently, machine learning is an important tool in the field of data science, and is becoming more important in biomedical research. This research also used artificial neural networks, which are a subfield of machine learning. Neural networks are composed of node layers, and input, one or more hidden layers, and an output layer [87,88,89]. In this study, we used a basic neural network to produce reliable results. This proof-of-concept exercise based on gene expression of celiac disease highlighted many markers, some known and other news.

Apart from *BTLA*, other markers were noted.

*CASP3*, caspase-3, belongs to the apoptotic signaling process and it is responsible for executing apoptosis. In celiac disease, apoptosis is an important mechanism for the epithelial and villous atrophy [90,91].

*PRDM1*, PR domain zinc finger protein 1, also known as BLIMP-1, is a transcription factor that mediates the function of T and NK cells in innate and adaptive immune responses. It also drives the maturation of B lymphocytes into immunoglobulin secreting cells (plasma cells) [92]. Plasma cells play an important role in the pathogenesis of celiac disease and are the most abundant gluten peptide MHC-expressing cells [93].

*GZMB*, granzyme B, is a protease present in the cytosolic granules of cytotoxic T lymphocytes (Tc) and natural killer (NK) cells, which activates caspase-independent pyroptosis into the target cells. In celiac disease, decreased expression of protease inhibitor 9, a GZMB inhibitor, is a potential mechanism of enterocyte destruction and villous atrophy [94].

*LAG3*, lymphocyte activation gene 3 protein, is an inhibitory receptor on antigen-activated T-cells [95]. It is present in type 1 T regulatory (Tr1) cells [96], which play a role in colitis [97]. Gliadin-specific type 1 regulatory T cells from the intestinal mucosa of treated celiac patients inhibit pathogenic T cells [98]. Endopeptidase mediated gliadin degradation by macrophages and concomitant IL-27 production drive differentiation of splenic gliadin-specific Tr1-like cells [99].

*STAT5A*, signal transducer and activator of transcription 5A, has dual functions including signal transduction and activation of transcription. STAT5A mediates cellular responses to cytokines and plays a role in homeostasis and in the function of innate lymphoid cells (ILCs) [100]. During gut inflammation, STAT5 promotes mucosal wound healing [101].

The classic celiac disease or gluten-sensitive enteropathy is clinically characterized by symptoms of malabsorption or diarrhea, histological changes in the small intestine consisting of villous atrophy, antibodies against tissue transglutaminase, and resolution following a gluten-free diet [1,102]. Additionally, there are other terms including atypical celiac disease, subclinical or asymptomatic disease, potential celiac disease, latent celiac disease, and refractory celiac disease [1]. The subtype of refractory celiac disease is of special interest because of the association with Enteropathy-Associated T-cell lymphoma (EATL) [103]. Nevertheless, this research focused on the “classic” variant or the “not otherwise specified (NOS)”.

In conclusion, this proof-of-concept exercise managed to model and predict celiac disease based on an autoimmune discovery panel; and highlighted pathogenic markers. Among these, we confirmed that celiac disease is characterized by increased BTLA expression.

## Figures and Tables

**Table 1:** Pathogenesis of Celiac Disease.

Factors	Pathophysiology	References
Dietary gluten	① Gluten of wheat, rye, and barley. Gliadins and glutenins are rich in proline, which makes them resistant to proteolysis by gastric and pancreatic enzymes. Various long gliadin peptides activate the immune system (“33mer”). Undigested peptides may also affect intestinal microbiota.	[18,19,20,21]
Genetics	① Genetic predisposition: HLA-DQ2 and HLA-DQ8 contribute to 20%–40% of the genetic risk. They are class II MHC expressed by antigen-presenting cells (APCs).	[22,23,24]
	② Forty-two non-HLA regions have been associated with celiac disease. It is estimated that they account for 15% of the genetic risk: *IL18R1*, *IL18RAP*, *STAT4*, *CD28*, *CTLA4*, *ICOS*, *CCR4*, *CCR1*, *CCR2*, *CCR3*, *CD3E*, *IL1R1*, *IL12A*, *IL2*, *IL21*, *TNFAIP3*, *ELMO1*, *PRKCQ*, *SOCS1*, *ICOSLG*, and *IRAK1*. These genes belong to cytokine-cytokine receptor activation, JAK-STAT pathway, T-cell receptor signaling, intestinal immune network for IgA production, NF-KB signaling, and cell adhesion molecules. Of note, many of these genes belong to the immune checkpoint and immune-oncology pathway.	[22,23,25,26,27,28]
Immune	① Generation of gluten-specific T-cell responses: presence of gluten-specific CD4-positive T lymphocytes, antibodies against gliadin and de enzyme TG2, and pro-inflammatory cytokines.	[29,30]
	② Generation of autoantibodies: activation and differentiation into plasma cells of gluten-specific and TG2-specific B lymphocytes, generation of autoantibodies that are both circulating and deposited in the mucosa. These autoantibodies are responsible for the increased permeability of the epithelial barrier.	[31,32,33]
	③ Cytokines in the intestinal mucosal immune system: IFN gamma and IL-21 are produced by gluten-specific CD4-positive T lymphocytes. Secretion of IL-15, IL-18, and inhibition of FOXP3-positive regulatory T lymphocytes (Tregs).	[34,35]
	④ Intraepithelial lymphocytes (IELs): increased in celiac disease and their amount correlates with mucosal atrophy. IELs display cytotoxic transformation and induce apoptosis of intestinal epithelial cells through FAS-L, perforin, granzyme B, and NKG2D. NKG2D interacts with MICA on epithelial cells.	[36,37,38,39,40,41,42]
	⑤ Innate immune activation: dysregulation of the production of IL-15 and activation of the innate immune response, including the induction of epithelial stress.	[43,44]
Environmental	① Microorganisms: intestinal dysbiosis (unbalanced intestinal microbiota) and increased prevalence of specific microbial virulence genes isolated from celiac disease patients.	[45,46,47,48,49,50]
	② Others, such as smoking	[51]

The pathogenesis of celiac disease is multifactorial and includes dietary gluten and genetic, immune, and environmental factors.

**Table 2:** Machine learning and artificial neural network analysis for predicting celiac disease.

Model	Overall Accuracy (%)	No. Genes (Fields) Used	Most Relevant Genes
C5	100	1	*IFNG*
Logistic regression	100	737	(Refer to Table 3)
Discriminant	100	737	-
LSVM ^1^	100	737	*CASP1*, *IL18*, *ARPC2*, *CASP3*, *KLF4*, *GBP1*, *SULT1A1*, *RNASET2*, *MIF*, and *PIGR*
SVM	100	737	-
XGBoost linear	100	737	-
XGBoost tree	100	737	-
CHAID	100	2	*BATF*, *GBP1*
C&R tree	100	6	*IFNG*
Random forest ^1^	100	737	*CXCL10*, *PRDM1*, *GZMB*, *STAT2*, *IL12RB1*, *LAG3*, *PTPN22*, *TMEM50B*, *IFI35*, *PRDX5*, *GALC*, *C1QBP*, *RIPK2*, *IFNG*, *CSF2*, *STAT5A*, *TNPO3*, *IQCB1*, and *DEXI*
Neural network ^1^	100	737	*CXCL2*, *IL7R*, *PLCH2*, *CCL23*, *MBD2*, *CSF3R*, *MUC1*, *GPR183*, *CD226*, and *PNMT*
KNN algorithm	96	737	-
Quest	96	6	*STAT1*
Random trees ^1^	86	737	*BTLA*, *CARD14*, *CASP10*, *CCL13*, *CCL5*, *CCR7*, *CXCL10*, *CXCL9*, *CXCR6*, *ELMO1*, and *EXTL*
Bayesian network	58	737	-

^1^ For LSVM, random forest, neural network and random trees, the genes are in order of importance for predicting celiac disease.

**Table 3:** Logistic regression.

Equation for Predicting Celiac Disease
−0.1765 × *AAMP* + −0.008 × *ABHD6* + −0.1178 × *ACKR2* + −1.725 × *ACOXL* + 0.6231 × *ACSL6* + 0.0009441 × *ADA* + 1.16 × *ADAM30* + 0.04882 × *ADCY3* + 1.108 × *ADCY7* + 0.2923 × *AFF3* + −0.5828 × *AGAP2* + 0.6009 × *AHI1* + 0.3013 × *AHR* + −0.002197 × *AIRE* + −0.7633 × *ANKRD55* + 0.06059 × *ANTXR2* + 0.2416 × *APEH* + 1.215 × *APOBEC3G* + −2.377 × *ARG1* + −0.2806 × *ARHGAP30* + 0.0796 × *ARID5B* + −0.0168 × *ARPC2* + −0.009025 × *ATF4* + −0.08039 × *ATG16L1* + −0.156 × *ATG5* + 0.09123 × *ATM* + 0.003949 × *B2M* + 0.02826 × *B3GNT2* + −0.2021 × *BABAM2* + 1.132 × *BACH2* + −0.6567 × *BAD* + −0.2759 × *BANK1* + −0.09905 × *BATF* + 0.617 × *BATF3* + 0.1081 × *BCL10* + −0.1113 × *BCL3* + 0.2034 × *BCL6* + 0.7125 × *BID* + 0.3596 × *BLK* + 0.1998 × *BLNK* + −0.4926 × *BORCS5* + −3.589 × *BSN* + 1.291 × *BTK* + −1.079 × *BTLA* + −1.254 × *BTNL2* + −0.1576 × *C1orf53* + 0.004046 × *C1QBP* + −49.65

## Data Availability

All the data, including methodology, are available upon reasonable request to Dr. Joaquim Carreras (joaquim.carreras@tokai-u.jp). The digital images and the list of 755 of the autoimmune discovery panel are uploaded to Zenodo platform, links: https://doi.org/10.5281/zenodo.6837120 and https://doi.org/10.5281/zenodo.6976192 (accessed on 9 August 2022).

## Supplementary Materials

The following supporting information can be downloaded at https://doi.org/10.5281/zenodo.6837120, Histological images of BTLA.

## Appendix A

The clinicopathological characteristics of the cases are shown in the Appendix A Table A1 including age, sex, biopsy location, diagnosis and the histological grade using the Marsh-Oberhuber classification [104,105].

**Table A1:** Clinicopatholgical characteristics.

Age	Sex	Biopsy Location	Diagnosis	Marsh-Oberhuber Classification
70	Male	Duodenum	Celiac Disease	3a
62	Male	Pylorus/duodenum	Celiac Disease/Chronic gastritis	2
62	Male	Duodenum	Celiac Disease	2
78	Female	Duodenum	Celiac Disease	3b
59	Male	Duodenum	Celiac Disease	3a
44	Female	Duodenum	Celiac Disease	2
17	Female	Duodenum	Celiac Disease	3b
56	Female	Duodenum	Celiac Disease	3a
54	Female	Duodenum	Celiac Disease	2
58	Female	Duodenum	Celiac Disease	3b
61	Female	Duodenum	Celiac Disease	3c
45	Male	Duodenum	Celiac Disease	3a
70	Female	Duodenum	Celiac Disease	2
40	Female	Duodenum	Celiac Disease	3a
61	Female	Duodenum	Celiac Disease	3c
44	Female	Duodenum	Celiac Disease	3a
